# Reliability analysis of the Manchester Triage System: inter-observer and intra-observer agreement^1^

**DOI:** 10.1590/1518-8345.2205.3005

**Published:** 2018-07-16

**Authors:** Cristiane Chaves de Souza, Tânia Couto Machado Chianca, Welfane Cordeiro, Maria do Carmo Paixão Rausch, Gabriela Fontoura Lana Nascimento

**Affiliations:** 2 PhD, Adjunct Professor, Departamento de Medicina e Enfermagem, Universidade Federal de Viçosa, Viçosa, MG, Brazil.; 3 PhD, Full Professor, Escola de Enfermagem, Universidade Federal de Minas Gerais, Belo Horizonte, MG, Brazil.; 4 MSc, President, Grupo Brasileiro de Classificação de Risco, Belo Horizonte, MG, Brazil.; 5 Specialist in Epidemiology in Health Services, Administrative Director, Grupo Brasileiro de Classificação de Risco, Belo Horizonte, MG, Brazil.; 6 RN, Grupo Brasileiro de Classificação de Risco, Belo Horizonte, MG, Brazil.

**Keywords:** Nursing, Emergency Medical Services, Triage, Nursing Assessment, Reproducibility of Results, Validity of Tests

## Abstract

**Objective:**

To analyze the reliability of the Manchester Triage System to determine the priority of patients in emergency services.

**Method:**

This is a reliability study with a sample of 361 nurses. The data were collected in three stages and the questionnaires were applied using the electronic software. The agreement was measured by the evaluation of clinical cases. The outcomes evaluated were agreement with the gold standard and intra-observer in the indication of the flowchart, discriminator, and level of risk. Data were submitted to univariate and bivariate analyses. The agreement was measured by the Kappa index.

**Results:**

The external and internal reliability of the protocol ranged from moderate to substantial (Kappa: 0.55-0.78). The time of professional experience as a nurse, in emergency services and in the classification of risk were associated with external and internal reliability. The correct choice of the discriminator influenced the correct indication of the risk level (R² = 0.77, p <0.0001) more than the correct choice of the flowchart (R² = 0.16, p <0.0001).

**Conclusion:**

The reliability of the Manchester Triage System ranged from moderate to substantial and it was influenced by the clinical experience of the nurse. The protocol is safe for defining clinical priorities using different classification flowcharts.

## Introduction

The triage process is an intuitive element of the clinical practice of nurses who work in emergency services. Assigning a degree of risk to the patient is a complex decision-making process. According to the Cognitive Continuum Theory, nurses use clinical reasoning to make decisions, which involves orderly and intentional thinking, based on theoretical and practical knowledge, and on personal and professional experience^1^. During the triage, decision-making is influenced by systems of support for clinical judgment, intuitive and reflexive judgment, and peer evaluation involving nurse and patient^2^.

The judgment support systems are the use of scales or triage systems that guide the nurse in evaluating the patient´s complaint. Intuitive and reflexive judgment is strongly influenced by the nurse´s professional experience, and by using previous experiences to judge new cases and make decisions. The peer evaluation involving nurses and patients implies the certification of the credibility of the complaint presented by the patient, involving him in the process of evaluation and confirmation of the clinical findings that support the decision on the level of priority^2^.

Regarding the support systems, triage systems or protocols have been developed to guide nurses´ evaluation^3^. Among them, the Manchester Triage System (MST) stands out, stratifying the severity levels by five and assigns the target color and time for medical care at each level. It is structured in flowcharts with discriminators that guide the collection and analysis of information to define the clinical priority of the patient^4^.

In a context of demand for services that is greater than supply and with limited assistance resources, patient triage must happen accurately to ensure care according to the patient´s real need^5^. Thus, nurses´ decision-making in risk classification should be guided by a reliable support system capable of accurately measuring the patient´s degree of priority.

Reliability is considered the main criterion for testing the quality of measuring instruments. It is the instrument´s ability to measure consistently and accurately what it is intended to measure and to reproduce a result consistently in time and space, or with different observers^6^.

Although MTS was developed two decades ago and it is widely used in different countries as a nurse support system for decision-making in triage, its reliability has been little questioned^7^. An integrative review has indicated that the reliability of the MTS ranged from moderate to near-perfect, showing the need for further studies to identify changes required in the protocol and to increase safety in the management of patients´ clinical risk in emergency departments^8^.

There are variations in the studies on the reliability of the MTS that indicate a gap on the real reliability of this triage scale^7^ ^-^ ^8^. Therefore, it is questioned: What is the reliability of the MTS to determine the degree of priority of the patient, considering the internal agreement and agreement between nurses who use this protocol? Thus, this study was designed to analyze the reliability of MTS to determine the priority of patients in emergency services.

## Method

This is a reliability study, conducted in partnership with the Brazilian Risk Classification Group (GBCR), which is responsible for the training of Brazilian nurses in the use and audit of MTS.

The study population consisted of nurses qualified by the GBCR between January 2008 and August 2014 to use the MTS in clinical practice (N=11,711). The qualification was obtained in the course of a classifier given by physicians and nurses of the GBCR, in the face-to-face modality, with duration of 12 hours. At the end, the nurses carried out a theoretical evaluation, and those that reached an index of achievement greater or equal to 60%, were considered apt to use STM in clinical practice. Nurses who had a valid electronic address and qualified in the classifier course (n=6,227 nurses) were included in the study.

The sample was calculated considering 95% confidence interval, 5% margin of error, an estimate of correct answers between observers and a gold standard of 75% and an estimate of intra-observer scores of 85%. The estimate correct answer used was based on a previously performed reference study^9^. Thus, to measure the external reliability of the MTS, the minimum sample required was 273 nurses and to measure the internal reliability, the minimum sample was 152 nurses.

However, it should be emphasized that the sample obtained in this study was higher than the minimum sample required. The final sample reached 361 nurses to measure the external reliability of the MTS, and there were 153 nurses to evaluate the internal reliability. They were nurses from 21 different Brazilian states, with the largest share of respondents from the states of Minas Gerais (93-25.76%), São Paulo (49-13.58%), Federal District (45-12.46%), Espírito Santo (45-12.46%), Rio Grande do Sul (31-8.59%), Ceará (26-7.20%), Santa Catarina (23-6.37%), and the rest (49-13.58%) of the states of Alagoas, Amazonas, Bahia, Goiás, Mato Grosso, Mato Grosso do Sul, Pará, Paraíba, Paraná, Pernambuco, Piauí, Rio de Janeiro, Rio Grande do Norte and Sergipe.

The reliability of the MTS was measured by the stability parameter, which consists of administering the same instrument to the same subjects under similar conditions on two or more occasions^10^. Clinical cases were evaluated by nurses at two different times (test and re-test).

Data collection was performed between September 2014 and August 2015, and it involved three stages. The first stage consisted of obtaining and validating clinical cases. A total of 40 cases were obtained from the GBCR, which corresponded to the total number of cases used at the time for the training of Brazilian nurses in the use of MTS. The cases were validated on the content by a group of three specialists qualified in the use of MTS in clinical practice and with publications on MTS, two with professional experience in the use of the protocol in clinical practice and one with experience in teaching and orientation of research involving MTS.

The version II of the Nursing Diagnostics Accuracy Scale (11) was used to validate the clinical cases, adapted for the study after the author´s consent to identify the degree that a set of clues described in each clinical case allowed the identification of the patient´s level of risk, following the MTS. For each clinical case, the specialists evaluated the presence, relevance, specificity, and coherence of the clues according to the clinical outcome (level of risk of the patient). The scale accuracy scores range from zero to 13.5, indicating from zero to high accuracy. Clinical cases were considered valid in which experts agreed with 100% that the clues described were of high accuracy (n=28).

In the second stage of the data collection, the test was performed to evaluate the external reliability of the MTS, measured by the agreement between the nurses and the gold standard in the indication, for each clinical case, of the flowchart, discriminator, and level of risk. The gold standard of the study was the template of clinical cases provided by GBCR. The SurveyGizmo® electronic software was used to collect the data with self-administered electronic questionnaires using computers connected to the Internet as a platform. The link to access the data collection instruments was sent to the e-mail of the study participants (n=6,227) through the MailChimp® tool. Upon accessing the link, the nurse was directed to the Informed Consent Form and, if he/she accepted to participate in the study, the nurse was directed to the home page, containing instructions on how to proceed to answer the questions. Two data collection instruments titled “Clinical cases” and “Professional profile of nurses” were configured in the SurveyGizmo^®^ system.

The instrument “Clinical cases” contained 28 validated clinical cases. For each clinical case, the nurse should indicate the flowchart, discriminator and level of risk using MTS. All responses were preformatted and no discursive responses were allowed. Clicking on the “Flowchart” field, the listing of the 50 flowcharts in the MTS was available. The two MTS flowcharts related to disaster response were excluded. By clicking on the chosen flowchart, the flowchart specific to that flowchart was available, as well as the definition of the discriminators, as it appears in the printed version of the MTS. Once the discriminator of the classification was chosen, the nurse indicated the patient´s level of risk.

The instrument “Professional profile of nurses” contained 13 questions grouped into two blocks: the questions related to the demographic and training profile of the nurse, and the questions related to the clinical experience of the nurse in nursing, emergency services, and in the risk classification.

In the third stage of the data collection, a re-test was performed to evaluate the internal reliability of MTS, measured by intra-observer agreement. The same data collection strategy described in stage two of the study was used. All nurses who answered the questionnaire in the second stage of data collection (n=361) were invited to participate in this stage of the study, responding again to the instrument “Clinical cases”, with the first classification being hidden.

The data collected were tabulated and submitted to univariate and bivariate analyses using the statistical program SPSS (Statistical Package for Social Sciences) version 19.0.

Nurses´ professional profile was used to describe descriptive statistics with a frequency distribution. To evaluate if the number of correct answers in the flowchart and discriminator indication influences the correct choice of level of risk, linear regression analysis was used. For this analysis, the dependent variable was the “number of correct answers in the choice of the risk level” and the independent variable was the “number of successes in choosing the flowchart” and the “number of correct answers in the discriminator’s choice”. It was also evaluated how the correct choice of the flowchart influenced the correct indication of the discriminator. For this analysis, the dependent variable was the “number of correct answers in the discriminator´s choice” and the independent variable was the “number of correctness in choosing the flowchart”.

The undertriage and overtriage values translating respectively the percentage of patients screened by nurses for levels of lower and higher severity, when compared to the gold standard, were measured using descriptive statistics with the calculation of the % of agreement between the nurses and the gold standard in choosing the level of risk.

The external and internal reliability of the MTS was assessed through the linear and unweighted Kappa agreement index, which measures intra-observer or inter-observer agreement beyond expected by chance, to which the same number of subjects were submitted. The agreement was considered: null (k=0); poor (0.01-0.19); weak (0.20-0.39); moderate (0.40 - 0.59); substantial (0.60 - 0.79), and almost perfect (0.80 - 1)^12^.

The Kruskal-Wallis non-parametric test was used to evaluate the association between the external and internal reliability of the MTS and the variables of the professional profile. For the cases of more than 2 groups where significant differences were found, the non-parametric test of Mann-Whitney with correction of Bonferroni was used.

The study was approved by the Ethics and Research Committee (Eight Opinion number 816,372) and followed the provisions of Resolution number 466/12 of the National Health Council on Research involving human beings.

## Results

Most of the 361 nurses who participated in the study (294 - 81.44%) were female, aged between 23 and 62 years old (mean: 34.16 years + 8.17 years). Table 1 shows the characterization of the nurses in the study, according to the variables of the professional profile investigated.

**Table 1:** Professional profile of study nurses (n=361). Belo Horizonte, MG, Brazil, 2015

Variable		n	%
Undergraduate Nursing time			
	Less than a year	2	0.55
	Between one and five years	144	39.89
	Between five and ten years	142	39.34
	More than ten years	73	20.22
Highest degree of training obtained or in progress			
	Graduation	40	11.08
	Specialization	276	76.46
	Master	33	9.14
	Doctorate degree	12	3.32
Training related to risk classification during graduation			
	No content	240	66.48
	Only theoretical content	90	24.93
	Theoretical and practical content	31	8.59
MTS-related training* during undergraduate			
	No contente	282	78.12
	Only theoretical content	60	16.62
	Theoretical and practical content with little or no application in clinical practice	16	4.43
	Theoretical and practical content with a lot of application in clinical practice	3	0.83
Time of professional experience as a nurse			
	Less than a year	30	8.31
	Between one and five years	164	45.43
	Between five and ten years	96	26.59
	More than ten years	71	19.67
Experience time in emergency services			
	Less than a year	82	22.71
	Between one and five years	188	52.08
	Between five and ten years	58	16.07
	More than ten years	33	9.14
Experience time in risk classification as a nurse			
	Never	40	11.08
	Less than a year	111	30.75
	Between one and five years	198	54.85
	Between five and ten years	11	3.05
	More than ten years	1	0.27

* MTS: Manchester Triage System

Table 2 shows the mean values and variations of the nurses’ correct answers with the gold standard in the indication of flowchart, discriminator and level of risk, considering the 28 validated clinical cases.

**Table 2:** Nurses´ correct answers with the gold standard in the indication of the flowchart, discriminator, and level of risk (n=361). Belo Horizonte, MG, Brazil, 2015

Variable	Number of correct answers			
	Average	Standar deviation	Maximum value	Minimum value
Flowchart	20.60	2.58	27	11
Discriminator	16.40	3.97	26	4
Level of risk	21.72	3.24	28	11

The linear regression analysis showed that there is a direct relationship between the number of correct answers of the flowchart and the discriminator and the indication of the level of risk (p <0.0001). The variation of correct answers in the choice of the flowchart explained 16% of the variation of the risk level (R² = 0.16, p <0.0001, 95% CI: 0.39-0.62), while the indication (R² = 0.77, p <0.0001, 95% CI: 0.67-0.76). The variation of correct answer in the choice of the flowchart explained 23% of the variation in the accuracy of the discriminators (R² = 0.23, p <0.0001, 95% CI: 0.60-0.88), proving the safety of the protocol in determining of the priority level from different presentation flowcharts, since there are complaints that can be evaluated using different presentation flowcharts.

In the assessment of the external and internal reliability of the MTS, the overtriage was more frequent in level V of severity (blue color), occurring in 17% to 18% of the cases. Undertriage was more frequent in level II of severity (orange color), occurring in 27% of the cases. The patients were screened to a level above and one level below the actual severity of the case.

The external and internal reliability of the MTS was measured by calculating the kappa index for the choice of the flowchart, discriminator, and risk level (Table 3).

For the choice of the flowchart and the level of risk, the MTS shows a substantial agreement. For the choice of the discriminator, the agreement is moderate. The investigation was carried out to verify the association between the kappa values and the variables of the professional profile. Table 4 shows the variables in which significant associations were found between the Kappa values and the choice of flowchart and classification discriminator in the evaluation of the external reliability of the MTS. No association was found between the variables of the professional profile and the agreement in the choice of the level of risk in the evaluation of the external reliability of the MTS.

**Table 3:** External (n=361) and internal (n=153) reliability of the Manchester Triage System: Kappa values. Level of significance: p <0.001. Belo Horizonte, MG, Brazil, 2015

Variable	External Reliability		Internal Reliability	
	Kappa: nursesand gold standard		Kappa intra-observer	Kappa: nursesand gold standard
Flowchart	0.72		0.78	0.73
Discriminator	0.55		0.57	0.59
Risk Level	0.69		0.70	0.72

**Table 4:** External Reliability of the Manchester Triage System: factors associated with an agreement between nurses and the gold standard in the choice of flowchart and discriminator (n=361). Belo Horizonte, MG, Brazil, 2015

Variable		Categories (Time)	Kappa Index			P-value*
			Mean	Standard Deviation	Median	
Flowchart Choice	Experience in emergency^‡^	Less than 1 year (a^†^)	0.70	0.10	0.70	0.04
		Between 1 and 5 years (a^†^,b^†^)	0.73	0.09	0.74	
		Between 5 and 10 years (b^†^)	0.74	0,10	0.74	
		More than 10 years (a^†^,b^†^)	0.71	0.08	0.70	
					
Choice of the Discriminator	Experience as a nurse^‡^	Less than 1 year (a^†^)	0.45	0.15	0.44	<0.001
		Between 1 and 5 years (a^†^,b^†^)	0.58	0.14	0.62	
		Between 5 and 10 years (b^†^)	0.56	0.15	0.58	
		More than 10 years (a^†^,b^†^)	0.52	0.13	0.55	
					
	Experience in emergency^‡^	Less than 1 year (a^†^)	0.48	0.13	0.51	<0.001
		Between 1 and 5 years (a^†^,b^†^)	0.52	0.17	0.51	
		Between 5 and 10 years (b^†^)	0.59	0.13	0.59	
		More than 10 years (a^†^,b^†^)	0.60	0.07	0.58	
					
	Experience in risk classification^§^	Never (b^†^)	0.50	0.12	0.53	<0.001
		Less than 1 year (b^†^)	0.55	0.15	0.57	
		Between 1 and 5 years (c^†^)	0.61	0.12	0.64	
		Between 5 and 10 years (c^†^)	0.62	0.06	0.60	
		More than 10 years (a^†^,c^†^)	0.40	--	0.32	

*P-value: p values obtained with the Kruskal-Wallis test. Level of significance: p<0.05.

†a, b, c: The letter “a”, “b” and “c” were used to name the groups compared in the Mann-Whitney test with Bonferroni correction. Equal letters symbolize equality between groups, and different letters reflect the differences found between groups.

‡ Experience in emergency and emergency, Nursing experience: Level of significance adopted for the Mann-Whitney test with Bonferroni correction for comparison of groups: p<0.006.

§ Experience in risk classification: Level of significance adopted for the Mann-Whitney test with Bonferroni correction for comparison of groups: p<0.0001.

When assessing the external reliability of the MTS to choose the classification flowchart, nurses with five to ten years of experience in emergency services presented a greater agreement with the gold standard, when compared to those with less than one year of experience, but, for both groups, an agreement was substantial (Table 4).

Nurses with less than one year of professional experience presented a lower agreement with the gold standard when compared to the other groups when choosing the discriminator. Similarly, nurses with less than one year of experience in emergency services agreed less to the gold standard in discriminator selection than those with one to five and five to ten years of experience, and the higher the time of experience, the higher the kappa values ​​found. There was no difference between those who had never acted and those who had acted for less than one year in the classification of risk and the agreement with the gold standard in the classification discriminator. However, those who work less than a year have been more compliant with the gold standard than those who have operated for more than ten years. Nurses with experience in the risk classification between five and ten years presented the highest values ​​of kappa with the gold standard in the discriminator choice (Table 4).

The internal reliability of the MTS was evaluated by 153 (42.4%) of the 361 nurses who evaluated the external reliability of the MTS, and the agreement found ranged from moderate to substantial (Table 3). Most nurses who participated in this stage of the study had between five years of professional experience as a nurse (43.8%), emergency services (58.2%), and risk classification (59.5%). Table 5 shows the variables in which significant associations between the Kappa values and the choice of the flowchart, discriminator, and level of risk were found.

**Table 5:** Internal Reliability of the Manchester Triage System: factors associated with an agreement in the choice of flowchart, discriminator and level of risk (n=153). Belo Horizonte, MG, Brazil, 2015

Variable		Categories (Time)	Kappa Index			P-value*
			Mean	Standard Deviation	Median	
Flowchart Choice	Experience in risk classification^‡^	Never (a^†^)	0.73	0.09	0.73	0.001
		Less than 1 year (a^†^)	0.75	0.09	0.77	
		Between 1 and 5 years (b^†^)	0.80	0.09	0.81	
		Between 5 and 10 years (a^†^,b^†^)	0.79	0.06	0.77	
					
Choice of the Discriminator	Experience in emergency^‡^	Never (a^†^)	0.48	0.14	0.51	0.001
		Less than 1 year (a^†^)	0.60	0.14	0.59	
		Between 1 and 5 years (b^†^)	0.58	0.11	0.55	
		Between 5 and 10 years (a^†^,b^†^)	0.51	0.04	0.51	
					
	Experience in risk classification^‡^	Never (a^†^)	0.52	0.14	0.53	<0.001
		Less than 1 year (a^†^)	0.51	0.12	0.53	
		Between 1 and 5 years (b^†^)	0.61	0.13	0.62	
		Between 5 and 10 years (a^†^,b^†^)	0.50	0.08	0.47	
						
Choice of risk level	Experience in emergency^‡^	Never (a^†^)	0.60	0.17	0.59	<0.001
		Less than 1 year (a^†^)	0.75	0.14	0.75	
		Between 1 and 5 years (b^†^)	0.69	0.11	0.70	
		Between 5 and 10 years (a^†^,b^†^)	0.60	0.16	0.64	
					
	Experience in risk classification^‡^	Never (a^†^)	0.63	0.15	0.65	<0.001
		Less than 1 year (a^†^)	0.62	0.15	0.61	
		Between 1 and 5 years (b^†^)	0.74	0.15	0.76	
		Between 5 and 10 years (a^†^,b^†^)	0.68	0.07	0.66	

*P-value: p values obtained with the Kruskal-Wallis test. Level of significance adopted: p<0.05.

† a, b: The letters “a” and “b” were used to name the groups compared in the Mann-Whitney test with Bonferroni correction. Equal letters symbolize equality between groups, and different letters reflect the differences found between groups.

‡ Experience in the classification of risk, experience in emergency and emergency: Level of significance adopted for the Mann-Whitney test with Bonferroni correction for comparison of groups: p <0.006.

The professional experience time influenced the internal agreement of the MTS. When choosing the classification flowchart, nurses with between one and five years of practical experience in risk classification presented near perfect agreement when compared to those less than one year of experience, or who never acted in the classification of risk. When choosing the discriminator, nurses who have between one and five years of experience in emergency services had higher average kappa values than those with less than one year of experience. Similarly, nurses with one to five years of risk rating experience had higher average kappa than those who had never performed, and those with less than one year of experience in this practice scenario (Table 5).

## Discussion

Most of the nurses who participated in this study (79.23%) had between one and ten years of undergraduate education, 66.48% said they had no content on risk classification during training, and 78.12% did not have any about MTS during graduation. The nurse integrates the evaluation of the complaint presented by the patient with his knowledge acquired during the training and the professional life to making the decision on the classification of risk, as well as the care environment in which he is inserted^5^. Thus, although it is necessary to be enabled by the GBCR to use MTS in clinical practice, it is recommended that content on the classification of risk and triage scales, especially the MTS, be included in the compulsory courses of training of nurses since graduation.

The triage scales or systems make up the decision support systems of nurses in the triage^2^. In this study, the reliability of the MTS was evaluated. The correct choice of the flowchart explained 16% of the variation in the correct indication of the level of risk, while the correct choice of the classification discriminator explained 77% of the variation in the correct indication of the risk level (p <0.0001). This finding shows that, as the MTS predicts, the nurse can choose similar flowcharts in the evaluation of a complaint, which will lead to the same level of risk, ensuring the safety of the protocol. This is because many complaints can lead to the choice of more than one presentation flowchart. Previous national and international studies that have proven this safety by statistical tests are not known.

Both in the evaluation of external reliability and in the evaluation of the internal reliability of the MTS, there were cases of overtriage and undertriage, being more frequent the cases of overtriage. A similar result was found in a meta-analysis, in which the frequency of overtriage using MTS was 46.65 and undertriage was 12.86%^5^. MTS is useful in the triage of patients in emergency services, but the classification of patients to levels above or below the actual level still occurs, being more frequent the cases of overtriage^13^. Screening patients above the correct priority level can lead to unnecessary use of resources in emergency services^14^. An underestimation of the level of risk may increase the risk of adverse consequences to patients, such as delayed care and the lack of adequate resources for their severity^15^.

The kappa values ​​found to indicate that the external and internal reliability of the MTS ranged from moderate to substantial, with slightly higher values ​​of kappa for intra-observer agreement (Table 3). A meta-analysis study conducted to assess the reliability of the MTS found that the overall agreement of the MTS was substantial (k=0.75). However, the authors emphasized that in the evaluated studies, the weighted kappa was used, which overestimate the reliability. Therefore, it is more prudent to consider that the current reliability of the MTS is moderate. In this study, the intra-observer agreement was also larger and near perfect, than the inter-observer agreement. The reliability among nurses was substantial (k=0.78), and among nurses and experts, it was almost perfect (k=0.86). The agreement was nearly perfect in the studies that measured it by the evaluation of patients in clinical practice (k=0.86) and it was substantial in the studies that evaluated agreement by the evaluation of clinical cases (k=0.76)^7^.

In an integrative review of the literature, the agreement between nurses and the gold standard using the MTS ranged from moderate to near perfect (k: 0.40 - 0.81), and intra-observer agreement ranged from substantial to near perfect (k: 0.65 - 0.84) (8). A large variation in inter-observer reliability was observed using the MTS, with kappa values between 0.31 (poor agreement) and 0.81 (near-perfect agreement), with the prevalence of studies that indicate a good to a very good agreement. The observed variation in kappa values was attributed to the differences in the studied populations, among the evaluators that use the MTS, and in the way, the MTS is applied in the studied places^14^.

A study conducted in Germany showed that agreement among nurses for the German version of the MTS was almost perfect (Kappa = 0.95)^16^. This is the highest agreement found in all studies available in the literature evaluating the reliability of the MTS. It should be noted that, unlike the Portuguese version of the MTS used in Brazil, the German version was submitted to a cultural adaptation process that resulted in language changes in the presentation flowcharts and in the definition of the discriminators. Thus, it is suggested to carry out a study that deals with the cultural adaptation and validation of MTS for use in Brazil to increase the reliability of the protocol.

Intuitive and reflexive judgment, components that involve decision-making in triage, is strongly influenced by nurses´ professional experience^2^. Corroborating this theory, in this study, the variables time of professional experience as a nurse, experience as a nurse in emergency services, and experience as a nurse in the risk classification were associated with the external and internal reliability of the MTS. In general, nurses between one and five years of experience and between five and ten years of experience obtained higher levels of agreement with each other and with the gold standard.

In Brazil, although there is an informal recommendation that the nurse should have previous experience in emergency services to act in the classification of risk, this is not a requirement regulated by the class council. In Italy, nurses are required to have at least six months´ experience in the triage patients in emergency services^17^.

Professional experience has been pointed out in the literature as a factor that influences nurses´ decision-making in triage. Nurses use previous knowledge and experience to make inferences and screen new cases^2^ ^,^ ^5^ ^,^ ^18^. The correct classification of risk depends on the training and experience of the nurse in the application of the MTS^15^.

It should be emphasized that the available literature is not conclusive about the amount of time of experience necessary to guarantee nurses´ competence in triage. This is the first Brazilian study that investigated and found an association between professional experience and the external and internal reliability of the MTS. In the study that evaluated nurses´ experience in the triage and the ability to use the MTS correctly, the Kappa values ​​between nurses and the gold standard were higher when the nurse´s experience with the MTS was higher, but no significant difference was found between the categories of experience in the triage analyzed^9^. The combination of the use of the MTS, the nurse´s experience in evaluating critically ill patients, and organizational factors accounted for 65% of safety in the correct patient triage. The experience of nurses contributes to greater patient safety than the triage system, which cannot completely replace the clinical skills that the experienced nurse has developed over the years in the profession^19^.

Thus, it is recommended to carry out new research to verify the association between the time of professional experience as a nurse, experience in emergency services, and experience in the classification of risk with the ability to use the MTS in clinical practice. It is also necessary to investigate the relationship between the time of experience in the use of the MTS and the ability to use it correctly in clinical practice.

Other studies have shown that the MTS exceeds its central objective, which is to establish priority for immediate treatment^13^ ^,^ ^20^ ^-^ ^21^.

A study carried out showed that the sensitivity of the MTS to identify patients who died or who needed hospitalization in intensive care units ranged from 0.80 to 0.86 in adults. The specificity to measure the same outcomes ranged from 0.84 to 0.91^13^. Patients classified as red, orange or yellow had a 4.86-fold greater risk of hospitalization, 5.58 times greater death, and a greater need to perform electrocardiogram and laboratory tests when compared to those classified as green or blue^20^. In Germany, a study showed that the higher the risk level of the patient, the longer the nursing time spent for patient care, showing that the MTS can be used to subsidize the calculation of the nursing staff in emergency services^21^. Thus, it can be seen that the MTS is able to predict early care needs in emergency services and to direct the organization of care and management of the clinic.

A limitation of the study was the use of clinical cases in software, to the detriment of the evaluation of patients in clinical practice or in a laboratory of realistic simulation with standardized patients. However, care for patients with clinical instability requires fast clinical evaluation and interventions at times determined according to the level of clinical priority, hindering to understand the data of interest in the study. It is known that the evaluation using standardized patients would provide greater realism and approximation with what occurs in clinical practice. However, collecting data using patient assessment in clinical practice or with standardized patients would restrict the number of nurses participating in the research and reduce them to a local sample. Thus, it was decided to work with a larger sample of nurses, in an attempt to generalize the results, and formatted the data collection instrument to reflect the decision-making process of the nurse using the MTS, approaching the clinical practice scenario. Nonetheless, prospective studies are suggested, using simulation methodology with standardized patients to better elucidate the reasons that lead to disagreements in the classification, as well as the elements that influenced nurses’ decision making.

Finally, it should be pointed out that the nurse´s role in the classification of risk is complex and that the decision-making involves, besides the cognitive elements, aspects such as the management of the flow of care and the organization of the care network, which extrapolate the power of governability of nurses. However, the use of a reliable instrument is important for nurses´ safety, considering that the protocol is their support system in decision making.

## Conclusion

The external and internal reliability of the MTS ranged from moderate to substantial, with Kappa values, respectively , between 0.55 and 0.72 (p <0.001) and between 0.57 and 0.78 (p <0.05). This was the first study that evaluated the external and internal reliability of the MTS for indication of the flowchart and the classification discriminator. The correct choice of the flowchart explained 16% of the variation in the correct indication of the risk level (R²: 0.16, p <0.0001), while the correct choice of the discriminator explained 77% of the correct choice of the risk level = 0.77, p <0.0001), confirming the safety of the MTS in determining the priority level from different flowcharts, since some patient complaints may lead to the choice of different presentation flowcharts.

In both the external and internal reliability evaluation of the MTS, there were cases of overtriage and undertriage and the disagreements occurred, respectively, to a level above, and to a level below the level of risk established as correct by the gold standard. It is recommended to carry out future studies that seek to understand the reasons for the disagreements to outline strategies aimed at increasing the reliability of the nurse´s evaluation in the use of the MTS.

Only the variables time of professional experience as a nurse, experience as a nurse in emergency services, and experience in the classification of risk were associated with the external and internal reliability of the MTS. In general, nurses between one and five years of experience and between five and ten years of experience obtained higher levels of agreement between themselves and the gold standard. These findings indicate that the insertion of nurses in clinical practice and previous experience in emergency services and in the classification of risk is important for the external and internal reliability of the MTS. However, considering the scarcity of available literature that evaluates the association between professional experience and the external and internal reliability of the MTS, it is early to establish a causal relationship between professional experience and the nurse´s accuracy in the use of the MTS. Thus, it is recommended to carry out new research to consolidate the findings of this study.
